# Biomedical engineering in low- and middle-income settings: analysis of current state, challenges and best practices

**DOI:** 10.1007/s12553-022-00657-8

**Published:** 2022-04-28

**Authors:** Carmelo De Maria, Andrés Díaz Lantada, Timo Jämsä, Leandro Pecchia, Arti Ahluwalia

**Affiliations:** 1 Research Center E. Piaggio and Dpt of Information Engineering, University of Pisa, Pisa, Italy; 2 Mechanical Engineering Dpt, Universidad Politecnica de Madrid, Madrid, Spain; 3 Physics and Technology, Research Unit of Medical Imaging, University of Oulu, Oulu, Finland; 4 Medical Research Center Oulu, University of Oulu and Oulu University Hospital, Oulu, Finland; 5 School of Engineering, University of Warwick, Coventry, CV4 7AL UK

**Keywords:** Biomedical engineering, Healthcare, Low- and middle-income settings, Medical technology

## Abstract

Supporting the expansion of best practices in Biomedical Engineering (BME) can facilitate pathway toward the providing universal health coverage and more equitable and accessible healthcare technologies, especially in low- and middle-income (LMI) settings. These best practices can act as drivers of change and may involve scientific-technological issues, human intervention during technology development, educational aspects, social performance management for improved interactions along the medical technology life cycle, methods for managing resources and approaches for the establishment of regulatory frameworks.

The aim of our study was to identify weaknesses and strengths of the scientific, technological, socio-political, regulatory and educational landscape in BME in LMI resource settings. We thus analysed the current state-of-the-art through six dimensions considered fundamental for advancing quality and equity in healthcare: 1) relevant and 2) emergent technologies, 3) new paradigms in medical technology development, 4) innovative BME education, 5) regulation and standardization for novel approaches, and 6) policy making. In order to evaluate and compare their relevance, maturity and implementation challenges, they were assessed through a questionnaire to which 100 professionals from 35 countries with recognized experience in the field of BME and its application to LMI settings responded.

The results are presented and discussed, highlighting the main challenges and pinpointing relevant areas where intervention, including local lobbying and international promotion of best practices is necessary. We were also able to identify areas where minimal effort is required to make big changes in global health.

**Supplementary information:**

The online version contains supplementary material available at 10.1007/s12553-022-00657-8.

## Introduction

The integration of science and technology, education and capacity building with the promotion of successful policies and international cooperation, are paramount to successfully approach the Sustainable Development Goals (SDG), and fulfil their objectives according to Agenda 2030 [1, 2]. In the specific area of “Good Health and Well-Being” (SDG 3), 13 targets and 698 proposed actions aim at “ensuring healthy lives and promoting well-being for all at all ages”. Amongst the most relevant data from the UN-SDGs website, less than half of the global population was covered by essential health services in 2017, and the COVID-19 pandemic has interrupted childhood immunization programmes in around 70 countries. Most targets within SDG 3 focus on minimizing maternal and child mortality, controlling epidemics, promoting healthy lifestyles, ensuring universal access to sexual and reproductive healthcare services and achieving universal health coverage. Biomedical Engineering (BME) and biomedical engineers and technicians play a fundamental role in attaining these targets, as healthcare technologies are necessary for modern medical practice, solving complex health issues, and in the long term, achieving health equity.

At scientific/research, professional/industrial and educational levels, BME has largely been a realm dominated by a few of the richest economies in the world. Nevertheless, some winds of change have been transforming BME as a field of study and action since the beginning of the 21st Century. The impacts of the connections between “Industry 4.0”, “Society 5.0” paradigms and healthcare [3, 4] are increasingly evident, even in low- and middle-income countries (LMICs), which constitute most of the world.

Nowadays, biomedical engineers are well-established professionals in almost every country, with varied roles such as medical technology developers, healthcare technology evaluators, regulators of sanitary products, health policy makers, technological supervisors at hospitals, and managers of healthcare systems [5]. BME study programmes are being taught around the world. Although universities from around 25 countries from Europe, North and South Americas and Asia fill the 300 first positions of the Shanghai ranking in BME, several new higher education programmes focused on biomedical or clinical engineering have also emerged in Africa, in the Middle East, and in Central and Southeast Asia [6]. Furthermore, the number of BME game-changing companies founded in countries without a long tradition in this field is steadily increasing and emerging economies are driving frugal innovation, through which truly transformative user-oriented medical technologies are achieved [7].

Healthcare technology equity has been put forward as a *sine qua non* condition to achieve universal healthcare coverage [8]. In fact, the World Health Organization (WHO) periodically prepares compendia on new and emerging medical technologies and priority medical devices and has even developed a global atlas of medical devices [9–11] to encourage dialogue between ministries of health, innovators, manufacturers, healthcare practitioners, users, and for stimulating the development of technologies for unsolved and urgent health problems. These reports show that the prevention, diagnosis, treatment and monitoring of all kinds of pathologies depend on further research as well as the development and deployment of innovative medical technologies. It is indeed widely accepted that donated medical technologies have been useless to address these problems, because they are not designed to work in remote regions and harsh environments and often contribute to transforming hospitals in developing countries into graveyards of medical technology [12, 13].

The entry on stage of SARS-CoV-2 and the COVID-19 pandemic are clearly demonstrating that our strategies and processes for globally solving unexpected problems must be improved. Indeed, the first pandemic wave highlighted that the world cannot rely on China as the world’s factory and that industrializing LMI settings and reindustrializing the EU are necessary, so that eventual bottlenecks do not prevent medical devices reaching those patients that need them most [14, 15]. The role of BME for achieving these transformations is evident.

In the authors’ opinion, collecting and supporting the expansion and sharing of best practices in the BME field can constitute a transformative strategy towards better health, through universal health coverage and more equitable and accessible medical technologies, especially in LMI settings. Best practices can be drivers of change – accelerating progress and inspiring role models – and may involve multiple dimensions including scientific-technological issues, hedonomics, learning/teaching approaches, management of physical and human resources, implementation of relevant regulations.

To this end, we devised a questionnaire to assess the current state-of-the-art of BME in LMI settings through the perceived impact, maturity and implementation challenges of its multifaceted dimensions. The questionnaire was administered to professionals with recognized experience in the field of BME and its application to LMI settings. By analysing the data through spider plots and performing cross-comparisons across the dimensions, we were able to pinpoint causes, effects and weak points in the healthcare landscape. This facilitated the identification of areas where intervention, including local lobbying and international promotion of best practices, is necessary, unnecessary, useful, harmful or indifferent.

This study offers a new perspective for defining priorities and outlining an action plan for progressing towards the 2030 Agenda leveraged by the integration of technological issues with social, educational and political challenges. We first detail the methodology used for the study, then present and discuss the main results, concluding with a series of recommendations for essential steps required to accelerate the achievement of SDG 3 targets.

## Methodology

### Questionnaire

The questionnaire was designed with the support of the IFMBE working group of “BME in Low- and Middle-Income Settings”, through a series of focus groups which met online regularly throughout 2019 and 2020.

We first identified six key dimensions within the scientific, technological, socio-political, regulatory and educational landscape in BME in LMI settings, which we considered foundational for advancing quality and equitable healthcare. The dimensions were: 1) state-of-the-art and 2) emergent technologies, 3) new paradigms in medical technology development, 4) innovative BME education, 5) regulation and standardization for novel approaches, and 6) policy making.

For each dimension, the questionnaire proposes a list of about 10 components, *e.g.,* technologies, actions, best practices, that characterize the dimension. For each component, a seven-point Likert scale was used for assessing its relevance (how important their role is in terms of potential for transforming healthcare), maturity (how mature or how well implemented they are nowadays) and difficulty (how challenging their implementation and sustainable promotion are) in the context of LMI settings. A value of 1 corresponds to an extremely irrelevant, immature, or easy to implement component; while a value of 7 corresponds to an extremely relevant, mature, or difficult to implement component. The combination of their relevance, maturity, and implementation difficulty can help identifying those components that could become effective key drivers of change.

Among technologies considered as “state-of-the-art” in richer economies, the questionnaire focuses on medical imaging resources, well-equipped surgical rooms, related minimally invasive technologies, autoclaving and sterilization devices, laboratories for microbiological testing and different technologies for supporting medical practice (prevention, diagnosis, therapy and monitoring) for varied population and age groups.

The dimension “emergent technologies” considers, for example, point-of-care microfluidic diagnostic systems, smartphone-based and AI-based technologies, virtual surgical training and robotic-aided surgery, the use of digital records and e-health tools, the use of 3D printing for medical device personalization, including tissue engineering and biofabrication, or innovative health affective technologies.

Besides analysing technologies, design methodologies together with their organizational and social aspects were also considered. New paradigms in medical technology development include collaborative design approaches and supporting (online) tools, co-creation with patients, healthcare professionals and end-users in general, point-of-care design and manufacturing options, design methods synergising with tools from Industry 4.0, donated and reprocessed medical technologies, to cite a few.

Education was also considered a fundamental dimension: the questionnaire covers educational aspects, including availability of educational materials and resources, existing BME programmes and accreditation systems, international mobility within LMI settings, supporting facilities, capacity building and training of educators, and innovative educational methods for resource-wise training.

Given the importance of normative frameworks and international cooperation, the development and application of standards and policy making were included as relevant dimensions. In particular, the questionnaire was designed to analyse opinions on the role and the readiness level of: harmonization of medical device regulation and nomenclature, availability of standards addressing the specificity of low resources settings, policies for promoting the local production of medical devices. The complete questionnaire is included in the Supplementary Information.

### Sample

**Fig. 1:**
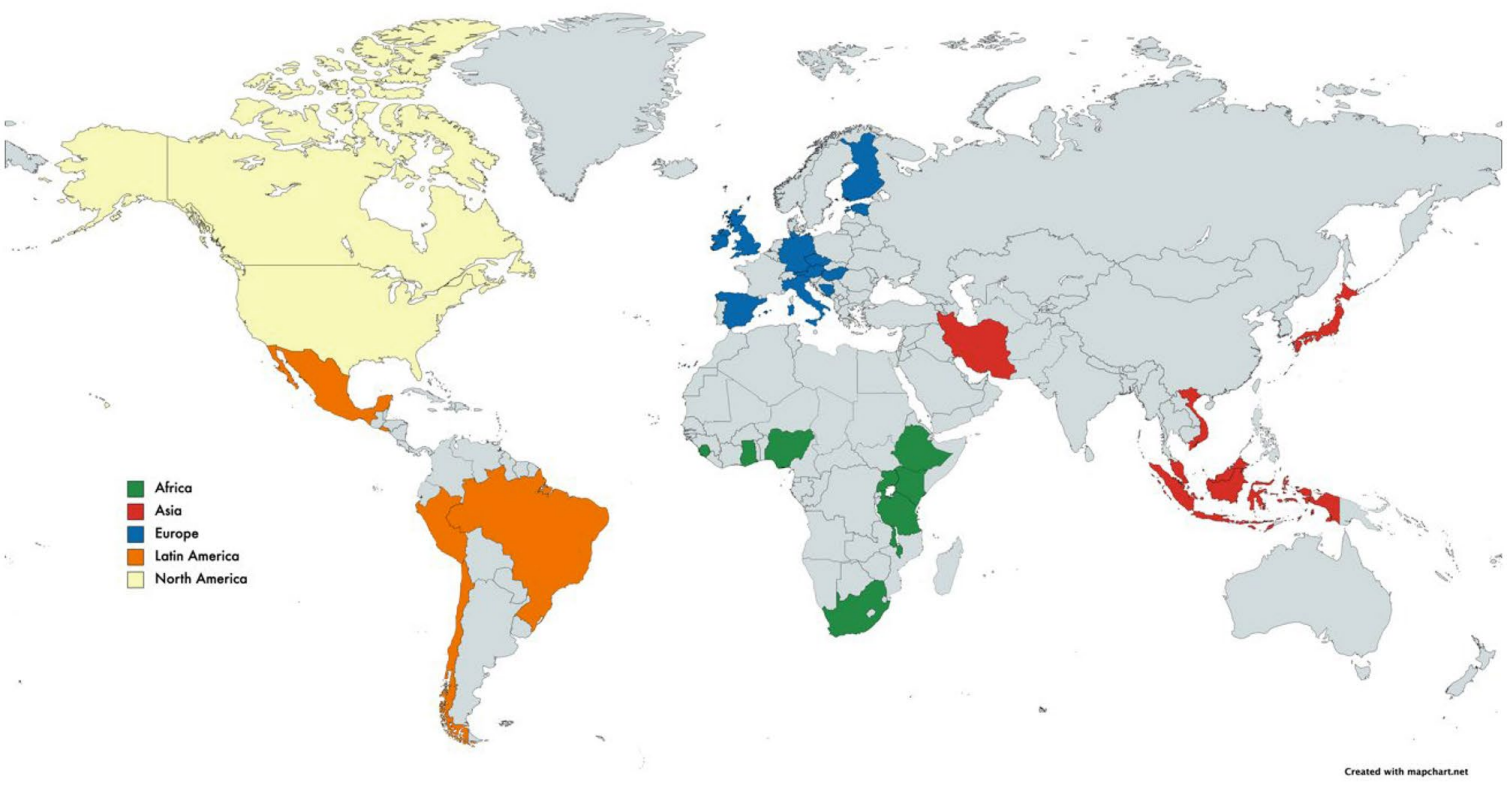
Geographical distribution of answers to the questionnaire

The questionnaire was implemented as an online survey using Google Forms, and open for answers for 3 months, from September 2020 to December 2020. Participants in the survey were reached through the support of the UBORA e-platform (http://ubora-biomedical.org), the International Federation of Medical and Biological Engineering (IFMBE, https://ifmbe.org/) together with all the federated national BME societies, the African Biomedical Engineering Consortium (ABEC, http://abec-africa.org), and the association Engineering for Change (https://www.engineeringforchange.org/). As shown in Fig. 1, 100 valid answers from 33 countries were received. Geographically the responses were from: Africa (21%, 9 countries), Asia (8%. 6 countries), Europe (55%, 11 countries), Latin America (5%, 5 countries), and North America (11%, 2 countries). The majority of respondents possess a PhD (52%), followed by a Master’s Degree (28%), a Bachelor’s Degree (11%) and Technician Diploma (9%). Of the respondents 49% work in Academia, 31% in Hospitals, 12% in Industry, 5% in NGO and 3% in Governmental Agencies. On average, they declare 18 years of experience, with however a very large standard deviation of 14 years.

### Statistical analysis

Data were analysed using Microsoft Excel, calculating descriptive statistics, including median and interquartile ranges. Aggregated data are expressed as a percentage of the total valid responses (including blanks and “I don’t know”) and provided in the Supplementary Information (Figs. A1-A6). The results are summarized in a spider graph, with relevance, maturity and feasibility as axes. In particular, feasibility was calculated as the complement of "difficulty” on a 7 step-scale (*i.e.*, feasibility = 7-difficulty). The normalized area of the spider graph of each component was calculated and named as “value index”, to create a ranking of the most promising technology/action/best practice for each dimension.

## Results and discussion

### State-of-the-art technologies: situation and challenges

State-of-the-art technologies listed in the study are all those resulting from the relevant biomedical engineering advances of the XX century, which are considered standard resources in well-equipped hospitals from the richer economies. These include most medical imaging equipment (*i.e.,* ultrasound, computed tomography, magnetic resonance imaging), well-equipped surgical rooms and related minimally invasive technologies (*i.e.,* catheters, endoscopes, electrosurgery tools, autoclaving and sterilization devices), laboratories for microbiological testing (PCR systems, laminar flow chambers, centrifuges), and different technologies for supporting medical practice for varied population and age groups (devices for prevention, diagnosis, therapy and monitoring and for safely storing medicines and vaccines).

**Fig. 2:**
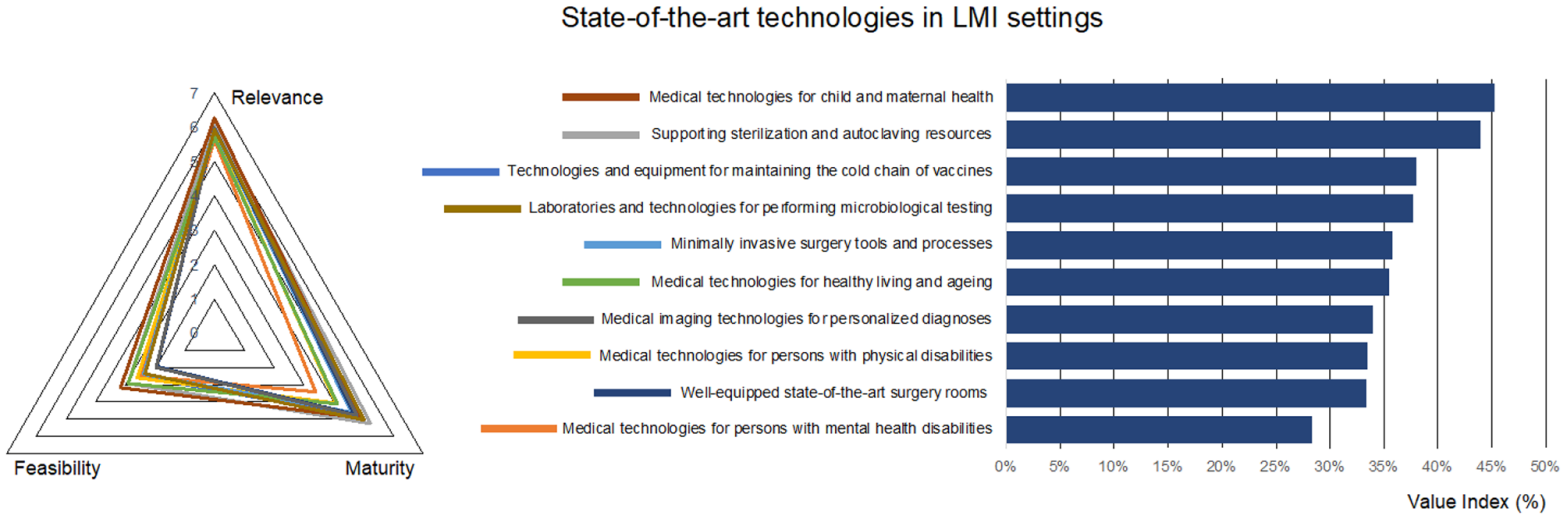
Prioritized technologies, actions, and best practices for the dimension “State-of-the-art technologies in LMI settings”

Based on the responses, medical technologies for child and maternal health are considered the most relevant for LMI settings (Fig. 2), in agreement with SDG 3 targets 3.1 and 3.2, which focus on maternal and child mortality, respectively. The maturity of these technologies is also considered fairly high as result of systematic actions in most countries resulting in recent improvements, as documented by WHO reports [16], although significant challenges remain.

Supporting sterilization and autoclaving resources are also considered fundamental, as they affect all surgical interventions and their maturity reaches the highest value of this dimension, considering that these are stable, robust, and affordable systems, as compared for instance with the more expensive and technology-intensive medical imaging systems. The latter are also relevant but seen as challenging to implement and still less mature, as corresponds to systems requiring relevant investment, accompanied by important operational and maintenance costs. Moreover, the need for specialized technicians for operating and interpreting the results from medical imaging systems underlines the importance of reinforcing medical and technical education, as well as the relevance of strategic planning for human resources.

Regarding well-equipped surgery rooms, they appear more mature, which may affect their being perceived as less relevant (or less urgent in this case, as is the case with the microbiological testing facilities). However, the more advanced minimally invasive surgical tools are seen as less relevant. They are also implemented to a limited extent, despite their benefits for minimizing the length of hospital stay and, hence, improving the allocation and management of resources. Possibly additional efforts for training surgeons and supporting personnel in these more advanced and less invasive techniques are required.

More concerning and surprising is the fact that technologies for healthy living and ageing and for the management of mental health are seen as the less relevant aspects of this dimension. Additionally, they are perceived to be the less mature, despite their easier resolution. As a result, these technologies have the lowest value index for this dimension. The modest relevance is especially concerning, considering that targets 3.4 and 3.5 of SDG 3 focus on minimizing the impact of non-communicable diseases, including improving mental health and the establishment of healthier living habits. In fact, the impact of unhealthy habits in the progression of diseases like diabetes, cancer, and respiratory diseases are expected to be the leading killers by 2030 in LMI settings [17]. Furthermore, mental health problems seem to be increasing, especially in Africa, and the number of people receiving adequate treatment is extremely low, according to a recent study [18]. The low perceived relevance may reflect that the problem is neglected or considered less urgent, and this may worsen the situation, affecting the fulfilment of the SDG 3 targets. Our results indicate that an urgent action plan, for improving healthy lifestyle habits and for promoting the relevance of mental health, is required. The implementation of systematic awareness campaigns, which are proving successful with the early detection of pathologies like cancer, can be part of the global strategy. In addition, trained psychiatrists are urgently needed.

### Emergent technologies: situation and challenges

Emerging technologies are defined as innovations characterized by radical novelty, fast growth, coherence, prominent impact, with some level of uncertainty and ambiguity in their outcomes and uses [19].

Being at the confluence of many disciplines, such as ICT, biology, medicine, and physics, BME has benefitted from innovations in other scientific areas (*e.g.*, smartphones, additive manufacturing) and has promoted the development of new enabling technologies, such as organ-on-chip and affecting computing. The following technologies, with potential impact in LMI settings were considered in the questionnaire: smartphone-based applications for monitoring patient and population health, including pandemic outbursts, personalized solutions for organ replacement through bioprinting or custom-made 3D printing medical devices, and ICT innovation including Artificial Intelligence (AI) and virtual reality.

**Fig. 3:**
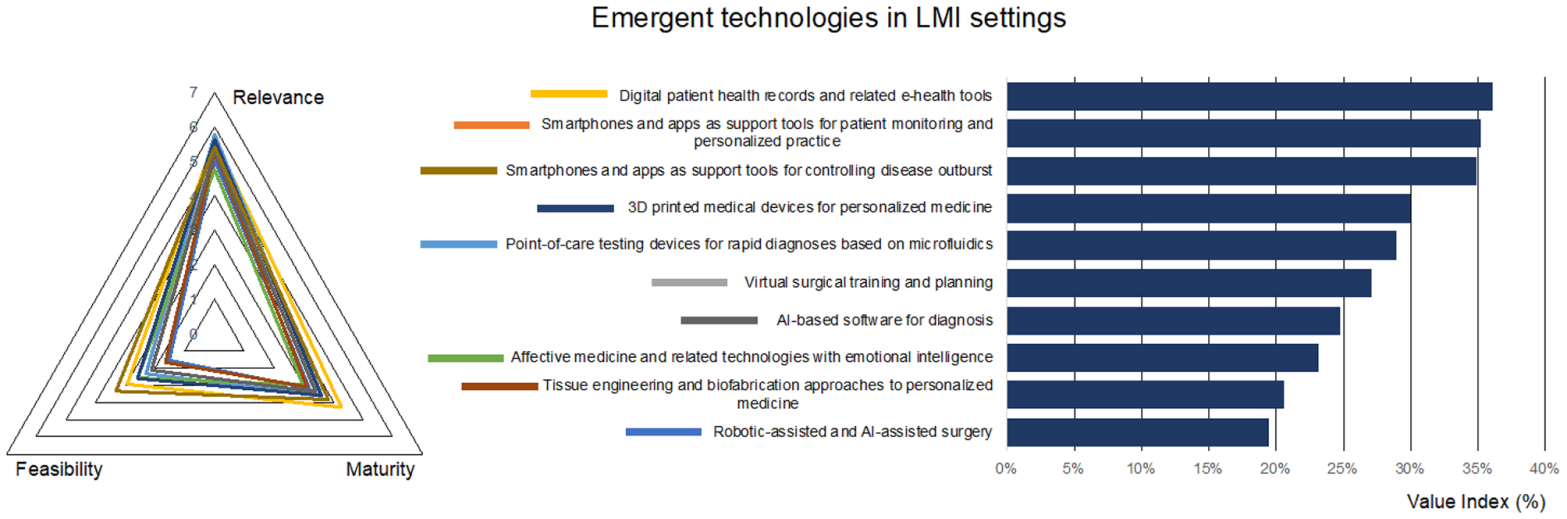
Prioritized technologies, actions, and best practices for the dimension “Emergent technologies in LMI settings”

According to the survey, digitalization of healthcare data has been indicated among the most relevant of actions with a good level of maturity (Fig. 3). Electronic Health and Medical Records (EHR and EMR) allow for robust dematerialization, easier maintenance, and continuous update. Despite persistent problems with data security, privacy regulations and interoperability, global strategies towards digital health are in place, even in countries with limited access to digital technologies [20, 21], where they are increasingly being used to facilitate good governance in the health sectors [22].

Tightly connected to data digitalization, mobile health is considered as adequately mature in LMI settings, which respect to other emergent technologies. The growing level of smartphone users (*e.g.*, in 2025 the 65% of the total population in Sub-Saharan Africa [23]) explains why mobile health solutions are considered relatively easy to implement in LMI settings. Despite the current hype on AI and its potential impact on SDGs [24], the results of the questionnaire do not assign a preeminent role to this technology in either diagnosis or applied to robotics (the lowest value index for this dimension, Fig. 3).

It has been argued that the perceived ability of emerging technologies to change the status quo could assume relevance in policy-making [19], indicating directions for investments from government and private foundations. In this context, affective computing, which is linked to mental health [18, 25], is considered by the panel of respondents as the less relevant and the less mature of technologies in LMI settings. This answer is consistent with that provided for state-of-the-art technologies for mental disabilities and suggests that mental health is not perceived as a priority in low-resources settings. A certain relevance is attributed to additive manufacturing, as an enabling technology for 3D printing personalized medical devices (*e.g.*, prosthesis) while, according to the results, the use of bioprinting technologies for tissue and organ replacement is far from realization. Indeed, even in high income countries, the technology is still immature and considered difficult to use or implement.

A final remark on microfluidic devices for rapid diagnostics, which were considered among the most relevant emergent technologies for LMI settings, even though they have yet to mature. The opinion is corroborated by the fact that rapid diagnostic tools are considered fundamental for pathologies such as malaria [26], and much effort has been directed to the promotion of paper-based microfluidics to support underserved rural communities [27].

### New approaches for the development of medical devices: situation and challenges

New approaches and strategies are needed to transform societies and healthcare, and these may affect the way in which medical technologies are developed. In this context, some of the components considered potentially transformative and analysed in the dimension “new approaches for the development of medical devices” of the questionnaire are: placing users in the forefront of technological development and benefiting from innovative cooperation and co-creation schemes; challenging the intellectual property *status quo* by resorting to open licensing options; bringing technology closer to patients, and rethinking supply chain management.

**Fig. 4:**
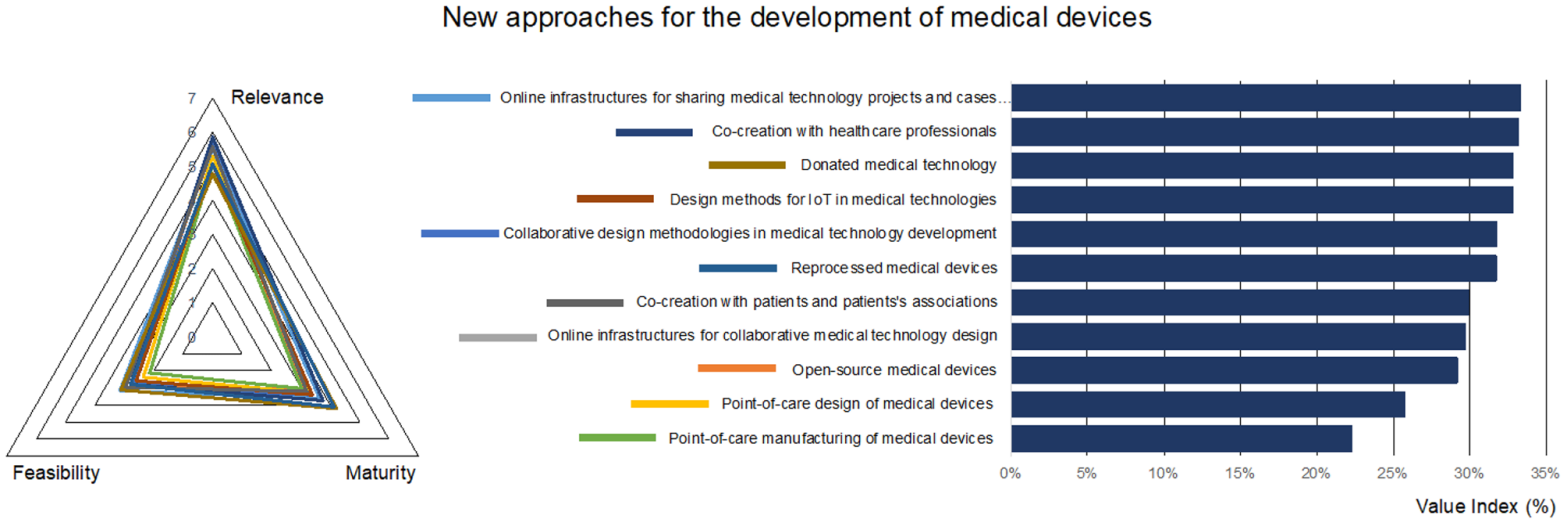
Prioritized technologies, actions, and best practices for the dimension “New approaches for the development of medical devices”

According to the responses, co-creation with healthcare professionals and patients and promotion of collaboration along the development life cycle of innovative medical technologies are the most valuable components of this dimension (Fig. 4). This clearly aligns with the proposals from well-validated systematic methodologies for successful medical product and technology development, which are progressively making users the protagonists of the conceptual stages for increased usability and safety [28, 29]. Human-centered design, design for usability, creative thinking, agile methods, lean innovation, are all different and synergic methods which can contribute to more straightforward design procedures leading to improved results.

These methods and approaches are also directly connected to the emergent field of open-source medical devices (OSMDs), wherein the development information is shared for increased health equity and as a way for promoting innovation in the medical field [30]. The recent efforts of researchers for fostering OSMDs are making a global impact. In fact, current open-source strategies and the related online platforms and resources for supporting co-creation and sharing of open-source solutions [31, 32] are considered by most respondents as the most relevant aspects of this dimension. These emergent innovative approaches are considered straightforward to implement, which bodes a promising future for this new trend related to the “makers’ movement” once it has gained maturity.

Donated medical technologies and refurbished medical equipment were considered as the least relevant aspects, although they are extremely common and mature (resulting in a high value index), thus worthy of consideration. Indeed, several studies report on the tendency of hospitals in LMI settings to become “graveyards of medical equipment and technologies” [12, 13, 33, 34]. Not only donations have a very low impact on the desired healthcare transformations, they end up having a detrimental affecting on the environment. There are several possible reasons why donated technology may not have the desired positive effects: firstly, donations are often based on old-fashioned technologies requiring increased maintenance; besides, the working conditions in LMI settings differ from those for which the technologies were originally designed; finally, once the systems breakdown, spare parts are hard to obtain, a problem worsened by the lack of trained technicians and the complex supply chains, among others. It could be argued that training biomedical engineers and building capacities for developing medical technologies in LMI settings is a much more useful and strategic approach, particularly in the long-term. These educational and capacity building actions may have an important contribution to the point-of-care design and manufacture of medical devices, which are considered both immature and challenging, with the lowest value indices.

### Innovative biomedical engineering education: situation and challenges

In BME or biomedical engineering technician (BMET) education, scientific-technological excellence is a prerequisite for designing, maintaining, repairing dedicated medical devices [35]. At the same time, attitude and competences for multidisciplinary collaborations and teamworking are also needed for developing user-centered and context-based devices [36], because catering to the specific social, cultural, and technological needs of a region have been considered one of the keys to a sustainable and efficient health care system [37, 38]. For these reasons, the fourth part of the questionnaire was dedicated to education, focusing on the relevance, maturity, and difficulty of online and physical sharing of and access to teaching/learning resources, internationalization, common accreditation systems, and doctoral and educator/teacher training.

**Fig. 5:**
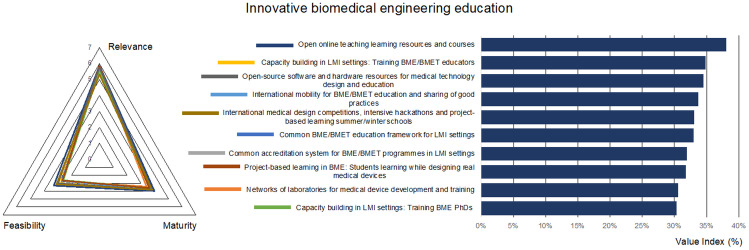
Prioritized technologies, actions, and best practices for the dimension “Innovative biomedical engineering education”

The online sharing of open educational resources (OERs) has been considered the most valuable route for innovate BME education in LMI settings (Fig. 5). An extensive list of OERs oriented to BME has been recently provided by Lantada and De Maria, covering teaching materials, hardware and software resources, and listing a series of active communities in the field [36]. Among these, we can cite UBORA, an open e-platform featuring project management tools for co-design of medical devices and teaching materials [31, 32].

International mobility and the sharing of good practices have been considered the most relevant and the most mature actions. Among the various exchange programmes, the European Union (EU) Programme Erasmus + , which promoted international mobility for higher education student and staff (including training for educators), is noteworthy [39]. The African Biomedical Engineering Mobility project is another example of such EU funded actions, promoting the mobility of African students among African high qualified institutions [40].

Project-based learning (PBL) methods have been indicated among the more relevant strategies for BME education, as proved by numerous initiatives [41–43]. However, these student-centered teaching–learning strategies have yet to become widespread to make an impact, as suggested by their perceived low maturity.

A common BME/BMET educational framework and accreditation systems, on the model of the Bologna process [44–46], have been considered quite relevant by the respondents, although not yet mature. To this scope, some activities have been put in place at a regional level to create a core-curriculum and share teaching resources in BME, for example in Latin America [47] or Africa [6]. In particular, ABEC was created with the endorsement of the United Nations Economic Commission for Africa (UNECA), with the goals of guiding innovation and improving health technology in Africa, facilitating information exchange and creation of learning platforms, and ensuring quality or standardization of BME university programmes at member institutions.

### Regulations and standards on medical devices: situation and challenges

The market of medical devices is regulated since these technologies may pose risks to operators and patients. As a consequence, in most countries, the efficacy, safety and risk benefit ratio of sanitary products or medical technologies must be evaluated before placing them on the market. It is important that regulations are not considered barriers to creativity or innovation, but necessary sets of legally binding documents and procedures that support medical device designers and manufacturers in their goals of transforming healthcare for the benefit of patients. However, the fact that regulations are not harmonized across countries, makes the certification path more complex and time-consuming, limits technological transfer and dramatically increases costs, all of which affects LMI settings more dramatically than in wealthier contexts [48].

Standards are technical documents aimed at promoting the sharing of good practices for more straightforward development of common technologies. Being sets of recommendations, their use is optional, although some especially relevant standards (e.g., ISO 10993, ISO 13485, ISO 14971, …), endorsed as “harmonized standards” by regulations such as the EU MDR 2017/745, have become almost compulsory for a streamlined approval by competent authorities [48].

In most cases, standards are developed by private organizations and, consequently, commercialized. A single standard can easily reach a price of 100–200 € and the development cycle of a common medical device may well benefit from the application of ca. 10 standards, which significantly increases the budget required for the innovation stages of the life cycle. This can constitute an additional barrier for innovators working in LMI settings.

**Fig. 6:**
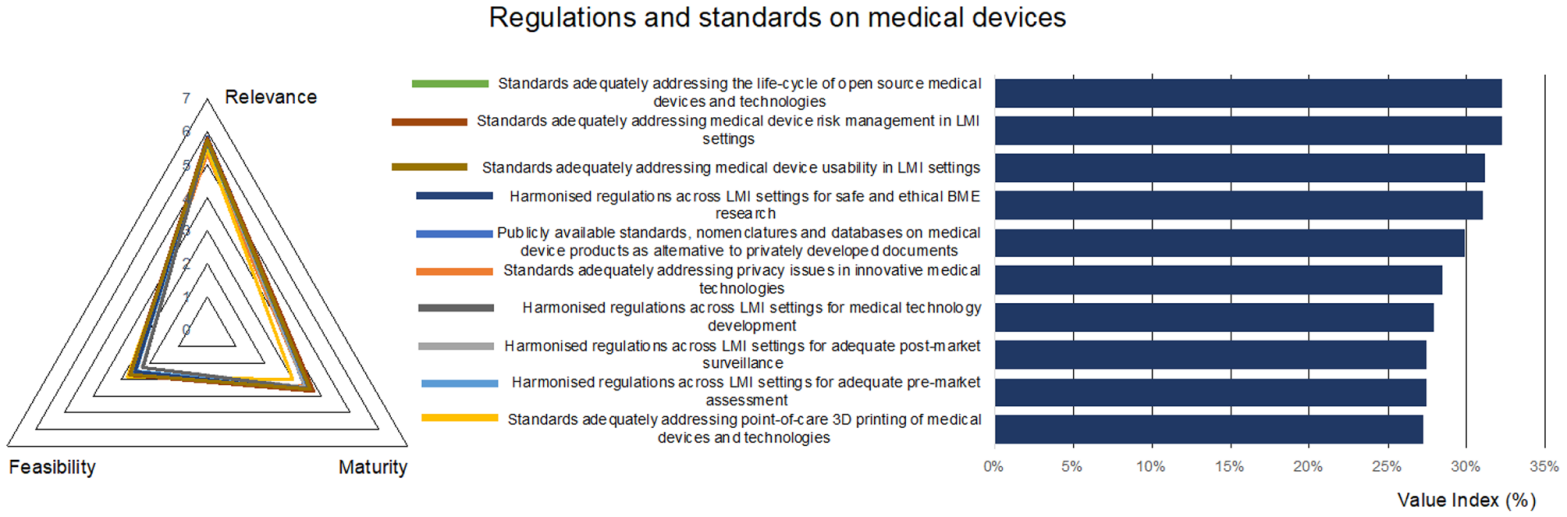
Prioritized technologies, actions, and best practices, for the dimension “Regulations and standards on medical devices”

From this dimension, the results show that the promotion of publicly available standards and the fostering of regulation harmonization across LMI settings are seen as the most relevant aspects to transform healthcare through technological equity, but the low maturity and the feasibility of these actions reduce their transformative potential (Fig. 6).

Most international standards are developed by national or international organizations, but normally involving representatives from the medical industry, from healthcare professionals, from regulatory bodies coming from richer countries. This leads to standards that do not properly consider the social, environmental and usability conditions in LMI settings, which makes many standards less universally applicable than they should be. The responses to our questionnaire highlight the need for developing standards specifically focusing on the usability and risk management in LMI settings. Notably, the development of specific standards for LMI setting is considered more straightforward to implement (highest value index for this dimension), than the international harmonization of regulations, which is probably conceived as more strategic and subject to political interests.

Regarding maturity, as compared with other dimensions of the questionnaire, it is important to underline that the harmonization of biomedical regulations across LMI settings is extremely immature. Worryingly, the “regulations and standards” dimension is globally seen as even less mature than the “emergent technologies” dimension, which focuses on the more *avant garde* biomedical resources and research trends. Clearly, efforts towards training experts in regulations and standards are needed, as well as additional strategic decisions and endeavours for international cooperation, including South-South cooperation schemes [49].

### Policy making and international partnerships: situation and challenges

Policy-making actions and international partnerships are fundamental aspects of the 2030 Agenda for sustainable development, so that the SDG 17 has been focused on a global partnership, where science and technology play a major role (Targets 17.6 and 17.8) [1, 2]. In this context, the questionnaire included an evaluation of the relevance, maturity, and difficulty in implementation of policies and working groups aimed at promoting capacity building in BME, new approaches in design, and production of medical technologies.

**Fig. 7:**
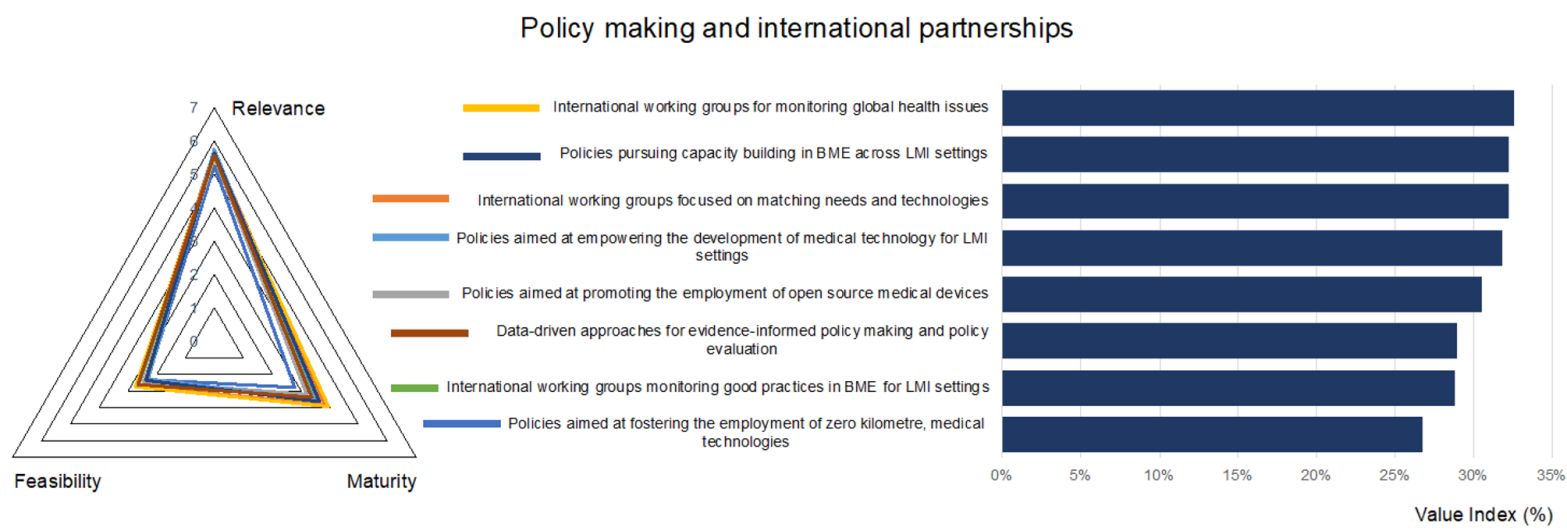
Prioritized technologies, actions, and best practices, for the dimension “Policy making and international partnerships”

In line with the results reported in Sect. 3.3 “New approaches for the development of medical devices”, the most relevant (but also the most difficult) policy is the empowerment of the development of medical technologies specifically for LMI settings (Fig. 7). In fact, only 10–30% of donated medical equipment becomes operational, given the high operating costs, the lack of personnel, and the frequent failures due to harsh environments, extreme climate conditions, humidity, dust, and power instability. None of these factors are considered during the design phase [30, 50–52]. On the other hand, the use of locally produced devices is not indicated as relevant, likely due to the unsolved issues related to safety and security, even in high income settings (lowest value index, Fig. 7).

Target 18 of SDG 17 is explicitly aimed at enhancing “capacity-building support to developing countries, including for least developed countries and small island developing States, to increase significantly the availability of high-quality, timely and reliable data disaggregated by income, gender, age, race, ethnicity, migratory status, disability, geographic location and other characteristics relevant in national contexts”. Considering the importance SDG 17 places on to e-health and data, the relevance of “data-driven policies” is not sufficiently valorised by our respondents.

The relevance of targeted working groups and tasks forces, but also their capacity to achieve results, is recognised in terms of maturity, despite the high difficulty of implementation. As example, the results of the Global Harmonization Task Force have provided a common basis towards the harmonization of medical device regulations [48]. The subsequent International Medical Device Regulators Forum is continuing its activity, providing guidance on conventional and cutting-edge technologies such as AI. Working groups for monitoring global health issues and good practices, but above all, matching needs and technologies are needed: to this end, virtual platforms and new approaches for co-design medical devices could support these activities.

## Conclusion: a pragmatic agenda

A questionnaire was designed to highlight the current state and the challenges of BME in low resource settings, with the final aim of identifying directions of actions for promoting health equity. Accordingly, the BME field was explored in 6 dimensions considered as pillars for advancement: mature and forthcoming technologies, design methodologies, education, regulations, and policy making. For each dimension, a selection of relevant components (*e.g.*, technologies, actions, best practices) was evaluated according to their perceived relevance, maturity, and feasibility in LMI settings, for highlighting those components which come closer to making a real impact and those which have yet to attain a relevant degree of maturity.

Considering the first two “components” for each dimension, a pragmatic agenda can be drafted including i) medical technologies for child/maternal health and for sterilization; ii) e-health and m-health; iii) sharing e-platforms for co-design with engineers and healthcare professionals; iv) OERs and capacity building for educators; v) standards addressing the new co-design methodologies and the specificity of LMI settings; iv) cross border actions both in monitoring health issues and the potential of new technologies. This agenda reflects some of the best practices currently in action in different low resources settings, some of which have been reported as complementary or synergic studies in the description and discussion of the outcomes of the questionnaire.

Interesting debates can be drafted analysing the components perceived as less relevant, mature or feasible, some of which reflect the common underestimation of a clinical need, such as mental health, or the obstacles to local production of safe medical technologies, or the difficulty for local training PhD students and transformative professional profiles.

Albeit limited in number, given the qualifications and experience of the participants, the results of this survey can be taken as a starting point for selecting future directions of research and health and education policies. Although a more global action should be pursued, in the authors’ opinion, starting with a modest contribution at local level, trying to address the suggested needs or following the identified best practices, may have tangible impacts within a short timeframe and lead to important or exemplary transformations in Biomedical Engineering.

## Supplementary Information

Below is the link to the electronic supplementary material.

Supplementary file1 (DOCX 98 KB)

Supplementary file2 (XLSX 37 KB)
