# Supplementary material for: Biomedical engineering in low- and middle-income settings: analysis of current state, challenges and best practices
Source: Health Technol (Berl). 2022 Apr 28;12(3):643–53. doi: 10.1007/s12553-022-00657-8 (PMC9045883; doi:10.1007/s12553-022-00657-8)
Supplement: Supplementary file 1 — Supplementary file1 (DOCX 98 KB) [file 12553_2022_657_MOESM1_ESM.docx]

**“Biomedical Engineering in Low- and Middle-Income Settings:**

**Analysis of Current State, Challenges and Best Practices”**

Supplementary material

**A.1. Questionnaire and results**

The following tables provide the descriptive statistics of the results of the questionnaire. Data are available also as spreasdsheet.

**State-of-the-art technologies in LMI settings**

|  |  | I don't  know | 1 (low) | 2 | 3 | 4 | 5 | 6 | 7 (high) | I  Quart. | III  Quart. | median | inter.  Range | mean | std |
| --- | --- | --- | --- | --- | --- | --- | --- | --- | --- | --- | --- | --- | --- | --- | --- |
| Medical imaging technologies for personalized diagnoses | Relevance | 6% | 2% | 0% | 4% | 5% | 16% | 19% | 47% | 5 | 7 | 7 | 2 | 6.0 | 1.4 |
|  | Maturity | 9% | 4% | 10% | 7% | 8% | 22% | 28% | 11% | 4 | 6 | 5 | 2 | 4.8 | 1.7 |
|  | Difficulty | 10% | 6% | 4% | 6% | 11% | 18% | 23% | 21% | 4 | 6 | 5 | 2 | 5.0 | 1.8 |
| Well-equipped state-of-the-art surgery rooms | Relevance | 8% | 0% | 2% | 6% | 8% | 8% | 18% | 49% | 5 | 7 | 7 | 2 | 6.0 | 1.4 |
|  | Maturity | 12% | 1% | 6% | 14% | 14% | 26% | 14% | 12% | 4 | 6 | 5 | 2 | 4.7 | 1.5 |
|  | Difficulty | 13% | 2% | 4% | 11% | 15% | 12% | 20% | 22% | 4 | 6.25 | 5 | 2.25 | 5.0 | 1.7 |
| Minimally invasive surgery tools and processes | Relevance | 7% | 1% | 1% | 8% | 3% | 13% | 18% | 48% | 5 | 7 | 7 | 2 | 5.9 | 1.4 |
|  | Maturity | 15% | 3% | 4% | 19% | 13% | 20% | 14% | 14% | 3 | 6 | 5 | 3 | 4.6 | 1.6 |
|  | Difficulty | 17% | 3% | 6% | 14% | 17% | 18% | 16% | 10% | 3 | 6 | 5 | 3 | 4.5 | 1.6 |
| Supporting sterilization and autoclaving resources | Relevance | 12% | 2% | 0% | 6% | 4% | 10% | 13% | 52% | 5.25 | 7 | 7 | 1.75 | 6.1 | 1.5 |
|  | Maturity | 19% | 1% | 1% | 9% | 14% | 16% | 19% | 21% | 4 | 6.5 | 5 | 2.5 | 5.2 | 1.5 |
|  | Difficulty | 20% | 7% | 5% | 13% | 25% | 12% | 13% | 5% | 3 | 5 | 4 | 2 | 4.1 | 1.6 |
| Technologies and equipment for maintaining the cold chain of vaccines | Relevance | 17% | 4% | 2% | 4% | 1% | 6% | 17% | 48% | 6 | 7 | 7 | 1 | 6.0 | 1.7 |
|  | Maturity | 29% | 3% | 4% | 9% | 14% | 13% | 10% | 16% | 4 | 6 | 5 | 2 | 4.8 | 1.7 |
|  | Difficulty | 31% | 7% | 5% | 7% | 11% | 15% | 10% | 13% | 3 | 6 | 5 | 3 | 4.5 | 1.9 |
| Laboratories and technologies for performing microbiological testing | Relevance | 17% | 2% | 3% | 4% | 3% | 11% | 16% | 44% | 5 | 7 | 7 | 2 | 5.9 | 1.6 |
|  | Maturity | 25% | 1% | 4% | 8% | 10% | 18% | 21% | 13% | 4 | 6 | 5 | 2 | 5.0 | 1.5 |
|  | Difficulty | 24% | 6% | 4% | 5% | 16% | 16% | 18% | 10% | 4 | 6 | 5 | 2 | 4.7 | 1.7 |
| Medical technologies for child and maternal health | Relevance | 16% | 0% | 1% | 4% | 1% | 10% | 15% | 52% | 6 | 7 | 7 | 1 | 6.3 | 1.2 |
|  | Maturity | 20% | 1% | 2% | 11% | 14% | 21% | 18% | 11% | 4 | 6 | 5 | 2 | 4.9 | 1.4 |
|  | Difficulty | 23% | 4% | 14% | 17% | 15% | 19% | 4% | 5% | 3 | 5 | 4 | 2 | 3.8 | 1.6 |
| Medical technologies for healthy living and ageing | Relevance | 5% | 3% | 2% | 3% | 10% | 17% | 17% | 42% | 5 | 7 | 6 | 2 | 5.7 | 1.6 |
|  | Maturity | 8% | 9% | 6% | 15% | 20% | 21% | 13% | 6% | 3 | 5 | 4 | 2 | 4.1 | 1.7 |
|  | Difficulty | 10% | 3% | 15% | 11% | 20% | 26% | 9% | 5% | 3 | 5 | 4 | 2 | 4.1 | 1.5 |
| Medical technologies for persons with physical disabilities | Relevance | 5% | 2% | 1% | 4% | 10% | 14% | 21% | 42% | 5 | 7 | 6 | 2 | 5.8 | 1.4 |
|  | Maturity | 11% | 6% | 11% | 13% | 18% | 24% | 13% | 3% | 3 | 5 | 4 | 2 | 4.1 | 1.6 |
|  | Difficulty | 10% | 6% | 9% | 10% | 16% | 18% | 24% | 6% | 3 | 6 | 5 | 3 | 4.4 | 1.7 |
| Medical technologies for persons with mental health disabilities | Relevance | 16% | 4% | 1% | 4% | 10% | 9% | 18% | 37% | 5 | 7 | 6 | 2 | 5.6 | 1.7 |
|  | Maturity | 23% | 11% | 16% | 11% | 16% | 12% | 8% | 1% | 2 | 5 | 3 | 3 | 3.4 | 1.6 |
|  | Difficulty | 23% | 8% | 7% | 3% | 14% | 16% | 17% | 10% | 4 | 6 | 5 | 2 | 4.5 | 1.9 |

**Emergent technologies in LMI settings**

|  |  | I don't  know | 1 (low) | 2 | 3 | 4 | 5 | 6 | 7 (high) | I  Quart. | III  Quart. | median | inter.  Range | mean | std |
| --- | --- | --- | --- | --- | --- | --- | --- | --- | --- | --- | --- | --- | --- | --- | --- |
| Point-of-care testing devices for rapid diagnoses based on microfluidics: | Relevance | 18% | 2% | 3% | 3% | 4% | 18% | 11% | 40% | 5 | 7 | 6 | 2 | 5.8 | 1.6 |
|  | Maturity | 25% | 9% | 14% | 10% | 19% | 11% | 7% | 4% | 2 | 5 | 4 | 3 | 3.6 | 1.7 |
|  | Difficulty | 27% | 0% | 9% | 7% | 12% | 24% | 8% | 12% | 4 | 6 | 5 | 2 | 4.7 | 1.6 |
| Virtual surgical training and planning: | Relevance | 11% | 6% | 1% | 4% | 10% | 19% | 18% | 30% | 5 | 7 | 6 | 2 | 5.4 | 1.7 |
|  | Maturity | 17% | 14% | 13% | 14% | 13% | 13% | 8% | 6% | 2 | 5 | 3 | 3 | 3.6 | 1.9 |
|  | Difficulty | 20% | 4% | 8% | 8% | 13% | 14% | 18% | 14% | 3.25 | 6 | 5 | 2.75 | 4.7 | 1.8 |
| Robotic-assisted and AI-assisted surgery: | Relevance | 10% | 6% | 8% | 6% | 11% | 13% | 13% | 32% | 4 | 7 | 5.5 | 3 | 5.0 | 2.0 |
|  | Maturity | 20% | 19% | 13% | 15% | 14% | 10% | 3% | 5% | 2 | 4 | 3 | 2 | 3.2 | 1.8 |
|  | Difficulty | 17% | 4% | 3% | 6% | 5% | 14% | 19% | 31% | 5 | 7 | 6 | 2 | 5.5 | 1.7 |
| AI-based software for diagnosis: | Relevance | 8% | 7% | 4% | 5% | 8% | 9% | 29% | 30% | 4 | 7 | 6 | 3 | 5.3 | 1.9 |
|  | Maturity | 13% | 14% | 15% | 18% | 18% | 9% | 7% | 5% | 2 | 4 | 3 | 2 | 3.4 | 1.7 |
|  | Difficulty | 16% | 6% | 3% | 10% | 10% | 15% | 23% | 17% | 4 | 6 | 5 | 2 | 4.9 | 1.8 |
| 3D printed medical devices for personalized medicine: | Relevance | 7% | 3% | 1% | 7% | 5% | 19% | 20% | 37% | 5 | 7 | 6 | 2 | 5.6 | 1.6 |
|  | Maturity | 11% | 11% | 11% | 20% | 20% | 16% | 7% | 3% | 2.25 | 5 | 4 | 2.75 | 3.6 | 1.6 |
|  | Difficulty | 11% | 6% | 8% | 10% | 18% | 21% | 15% | 10% | 3 | 6 | 5 | 3 | 4.4 | 1.7 |
| Affective medicine and related technologies with emotional intelligence | Relevance | 29% | 7% | 2% | 8% | 11% | 11% | 18% | 13% | 4 | 6 | 5 | 2 | 4.8 | 1.9 |
|  | Maturity | 37% | 17% | 10% | 14% | 7% | 8% | 5% | 1% | 1 | 4 | 3 | 3 | 3.0 | 1.7 |
|  | Difficulty | 40% | 6% | 2% | 11% | 11% | 10% | 9% | 10% | 3 | 6 | 4 | 3 | 4.4 | 1.8 |
| Digital patient health records and related e-health tools | Relevance | 6% | 3% | 6% | 4% | 5% | 15% | 15% | 44% | 5 | 7 | 6 | 2 | 5.6 | 1.7 |
|  | Maturity | 8% | 5% | 6% | 21% | 13% | 28% | 10% | 8% | 3 | 5 | 5 | 2 | 4.3 | 1.6 |
|  | Difficulty | 9% | 5% | 14% | 15% | 18% | 21% | 7% | 10% | 3 | 5 | 4 | 2 | 4.1 | 1.7 |
| Smartphones and apps as support tools for controlling disease outburst | Relevance | 10% | 3% | 4% | 9% | 6% | 12% | 23% | 32% | 4.75 | 7 | 6 | 2.25 | 5.4 | 1.7 |
|  | Maturity | 12% | 14% | 11% | 6% | 16% | 27% | 8% | 4% | 2 | 5 | 4 | 3 | 3.8 | 1.8 |
|  | Difficulty | 12% | 10% | 13% | 17% | 19% | 17% | 5% | 6% | 2 | 5 | 4 | 3 | 3.7 | 1.7 |
| Smartphones and apps as support tools for patient monitoring and personalized practice | Relevance | 7% | 3% | 6% | 1% | 8% | 24% | 19% | 31% | 5 | 7 | 6 | 2 | 5.4 | 1.6 |
|  | Maturity | 10% | 8% | 13% | 14% | 23% | 16% | 13% | 4% | 3 | 5 | 4 | 2 | 3.9 | 1.6 |
|  | Difficulty | 9% | 6% | 13% | 21% | 26% | 15% | 4% | 5% | 3 | 5 | 4 | 2 | 3.7 | 1.5 |
| Tissue engineering and biofabrication approaches to personalized medicine | Relevance | 16% | 7% | 4% | 7% | 6% | 11% | 15% | 34% | 4 | 7 | 6 | 3 | 5.3 | 2.0 |
|  | Maturity | 24% | 21% | 6% | 19% | 15% | 8% | 5% | 2% | 1 | 4 | 3 | 3 | 3.1 | 1.7 |
|  | Difficulty | 26% | 7% | 0% | 4% | 7% | 12% | 16% | 27% | 4.75 | 7 | 6 | 2.25 | 5.3 | 1.9 |

**New approaches for the development of medical devices**

|  |  | I don't  know | 1 (low) | 2 | 3 | 4 | 5 | 6 | 7 (high) | I  Quart. | III  Quart. | median | inter.  Range | mean | std |
| --- | --- | --- | --- | --- | --- | --- | --- | --- | --- | --- | --- | --- | --- | --- | --- |
| Collaborative design methodologies in medical technology development | Relevance | 14% | 3% | 3% | 3% | 5% | 13% | 18% | 40% | 5 | 7 | 6 | 2 | 5.8 | 1.6 |
|  | Maturity | 19% | 13% | 6% | 19% | 20% | 12% | 4% | 7% | 3 | 5 | 4 | 2 | 3.6 | 1.8 |
|  | Difficulty | 20% | 6% | 5% | 13% | 22% | 17% | 6% | 11% | 3 | 5 | 4 | 2 | 4.3 | 1.7 |
| Online infrastructures for collaborative medical technology design | Relevance | 13% | 6% | 3% | 0% | 7% | 21% | 14% | 35% | 5 | 7 | 6 | 2 | 5.5 | 1.8 |
|  | Maturity | 17% | 16% | 13% | 16% | 19% | 10% | 4% | 6% | 2 | 4.25 | 3 | 2.25 | 3.4 | 1.8 |
|  | Difficulty | 17% | 9% | 5% | 12% | 19% | 19% | 9% | 9% | 3 | 5 | 4 | 2 | 4.2 | 1.8 |
| Online infrastructures for sharing medical technology projects and cases of success | Relevance | 9% | 4% | 4% | 3% | 8% | 16% | 19% | 36% | 5 | 7 | 6 | 2 | 5.5 | 1.7 |
|  | Maturity | 13% | 11% | 13% | 18% | 18% | 11% | 7% | 7% | 2 | 5 | 4 | 3 | 3.6 | 1.8 |
|  | Difficulty | 14% | 7% | 10% | 20% | 22% | 14% | 5% | 8% | 3 | 5 | 4 | 2 | 3.8 | 1.7 |
| Co-creation with healthcare professionals | Relevance | 10% | 4% | 2% | 3% | 5% | 8% | 22% | 45% | 5.25 | 7 | 6.5 | 1.75 | 5.9 | 1.6 |
|  | Maturity | 14% | 11% | 6% | 18% | 25% | 13% | 7% | 5% | 3 | 5 | 4 | 2 | 3.8 | 1.6 |
|  | Difficulty | 15% | 5% | 9% | 18% | 15% | 18% | 6% | 13% | 3 | 5 | 4 | 2 | 4.2 | 1.8 |
| Co-creation with patients and patients's associations | Relevance | 15% | 5% | 2% | 6% | 4% | 12% | 18% | 38% | 5 | 7 | 6 | 2 | 5.6 | 1.8 |
|  | Maturity | 22% | 16% | 13% | 19% | 14% | 11% | 2% | 4% | 2 | 4 | 3 | 2 | 3.2 | 1.7 |
|  | Difficulty | 24% | 6% | 11% | 16% | 11% | 15% | 7% | 10% | 3 | 5 | 4 | 2 | 4.0 | 1.8 |
| Open-source medical devices* | Relevance | 10% | 7% | 3% | 5% | 5% | 8% | 17% | 44% | 5 | 7 | 6 | 2 | 5.6 | 1.9 |
|  | Maturity | 18% | 21% | 13% | 22% | 8% | 8% | 3% | 7% | 1.75 | 4 | 3 | 2.25 | 3.1 | 1.9 |
|  | Difficulty | 19% | 9% | 8% | 16% | 13% | 15% | 11% | 9% | 3 | 5 | 4 | 2 | 4.0 | 1.9 |
| Point-of-care design of medical devices** | Relevance | 8% | 7% | 1% | 8% | 9% | 15% | 16% | 34% | 4 | 7 | 6 | 3 | 5.3 | 1.9 |
|  | Maturity | 16% | 18% | 13% | 16% | 13% | 13% | 6% | 4% | 2 | 5 | 3 | 3 | 3.3 | 1.8 |
|  | Difficulty | 15% | 7% | 7% | 6% | 16% | 14% | 18% | 16% | 3.75 | 6 | 5 | 2.25 | 4.6 | 1.9 |
| Point-of-care manufacturing of medical devices*** | Relevance | 8% | 6% | 3% | 10% | 11% | 15% | 18% | 28% | 4 | 7 | 5.5 | 3 | 5.1 | 1.9 |
|  | Maturity | 14% | 20% | 18% | 15% | 18% | 6% | 7% | 2% | 2 | 4 | 3 | 2 | 3.0 | 1.7 |
|  | Difficulty | 14% | 4% | 8% | 6% | 15% | 16% | 16% | 21% | 4 | 6 | 5 | 2 | 4.9 | 1.8 |
| Design methods for IoT in medical technologies | Relevance | 25% | 6% | 2% | 6% | 9% | 15% | 13% | 23% | 4 | 7 | 5 | 3 | 5.1 | 1.9 |
|  | Maturity | 30% | 14% | 14% | 9% | 11% | 14% | 3% | 5% | 2 | 5 | 3 | 3 | 3.4 | 1.8 |
|  | Difficulty | 31% | 3% | 13% | 3% | 15% | 16% | 7% | 12% | 3 | 6 | 5 | 3 | 4.4 | 1.8 |
| Donated medical technology | Relevance | 19% | 9% | 4% | 7% | 11% | 14% | 13% | 23% | 3.25 | 7 | 5 | 3.75 | 4.8 | 2.0 |
|  | Maturity | 27% | 7% | 10% | 11% | 10% | 9% | 14% | 10% | 3 | 6 | 4 | 3 | 4.2 | 1.9 |
|  | Difficulty | 25% | 9% | 13% | 13% | 9% | 12% | 12% | 7% | 2 | 5.5 | 4 | 3.5 | 3.9 | 1.9 |
| Reprocessed medical devices | Relevance | 22% | 6% | 4% | 7% | 7% | 11% | 19% | 23% | 4 | 7 | 6 | 3 | 5.1 | 1.9 |
|  | Maturity | 27% | 6% | 9% | 10% | 14% | 13% | 14% | 5% | 3 | 6 | 4 | 3 | 4.1 | 1.7 |
|  | Difficulty | 25% | 5% | 13% | 9% | 14% | 13% | 9% | 12% | 3 | 6 | 4 | 3 | 4.2 | 1.9 |

* devices developed collaboratively, whose complete project information is made accessible for free

** medical technologies designed in the actual point-of-care, either in engineering labs within hospitals or in facilities nearby involving local population

******* medical technologies manufactured in the actual point-of-care, either in labs within hospitals or in facilities nearby involving local population

**Innovative biomedical engineering education**

|  |  | I don't  know | 1 (low) | 2 | 3 | 4 | 5 | 6 | 7 (high) | I  Quart. | III  Quart. | median | inter.  Range | mean | std |
| --- | --- | --- | --- | --- | --- | --- | --- | --- | --- | --- | --- | --- | --- | --- | --- |
| Common BME/BMET education framework for LMI settings | Relevance | 10% | 4% | 0% | 4% | 5% | 15% | 17% | 44% | 5 | 7 | 6 | 2 | 5.8 | 1.6 |
|  | Maturity | 15% | 9% | 13% | 18% | 13% | 21% | 7% | 5% | 2.25 | 5 | 4 | 2.75 | 3.8 | 1.7 |
|  | Difficulty | 14% | 6% | 8% | 15% | 12% | 23% | 12% | 8% | 3 | 5 | 5 | 2 | 4.3 | 1.7 |
| Common accreditation system for BME/BMET programmes in LMI settings | Relevance | 14% | 4% | 2% | 6% | 7% | 14% | 15% | 36% | 5 | 7 | 6 | 2 | 5.5 | 1.7 |
|  | Maturity | 24% | 8% | 14% | 8% | 16% | 12% | 8% | 8% | 2 | 5 | 4 | 3 | 3.9 | 1.9 |
|  | Difficulty | 23% | 5% | 6% | 10% | 19% | 20% | 10% | 7% | 3 | 5 | 4 | 2 | 4.3 | 1.6 |
| International mobility for BME/BMET education and sharing of good practices | Relevance | 12% | 3% | 1% | 4% | 3% | 14% | 19% | 43% | 5 | 7 | 6 | 2 | 5.9 | 1.5 |
|  | Maturity | 19% | 8% | 10% | 13% | 15% | 18% | 9% | 7% | 3 | 5 | 4 | 2 | 4.0 | 1.8 |
|  | Difficulty | 19% | 5% | 8% | 14% | 12% | 15% | 16% | 9% | 3 | 6 | 5 | 3 | 4.4 | 1.8 |
| Open online teaching learning resources and courses | Relevance | 3% | 3% | 2% | 8% | 1% | 13% | 19% | 51% | 5 | 7 | 7 | 2 | 5.9 | 1.6 |
|  | Maturity | 8% | 11% | 10% | 20% | 11% | 17% | 10% | 11% | 3 | 5 | 4 | 2 | 4.0 | 1.9 |
|  | Difficulty | 8% | 11% | 11% | 19% | 20% | 17% | 10% | 3% | 2.75 | 5 | 4 | 2.25 | 3.7 | 1.6 |
| Open-source software and hardware resources for medical technology design and education | Relevance | 8% | 3% | 2% | 8% | 3% | 10% | 22% | 43% | 5 | 7 | 6 | 2 | 5.8 | 1.6 |
|  | Maturity | 14% | 11% | 10% | 18% | 16% | 13% | 7% | 9% | 2.75 | 5 | 4 | 2.25 | 3.8 | 1.8 |
|  | Difficulty | 16% | 8% | 13% | 9% | 19% | 19% | 10% | 6% | 3 | 5 | 4 | 2 | 4.0 | 1.7 |
| Networks of laboratories for medical device development and training | Relevance | 10% | 4% | 1% | 5% | 1% | 15% | 20% | 43% | 5 | 7 | 6 | 2 | 5.8 | 1.6 |
|  | Maturity | 21% | 11% | 16% | 15% | 14% | 13% | 2% | 6% | 2 | 5 | 3 | 3 | 3.4 | 1.7 |
|  | Difficulty | 19% | 8% | 6% | 7% | 16% | 23% | 17% | 4% | 3 | 5.75 | 5 | 2.75 | 4.3 | 1.7 |
| Capacity building in LMI settings: Training BME/BMET educators | Relevance | 16% | 2% | 2% | 4% | 4% | 13% | 20% | 38% | 5 | 7 | 6 | 2 | 5.8 | 1.5 |
|  | Maturity | 25% | 5% | 9% | 22% | 8% | 21% | 5% | 5% | 3 | 5 | 4 | 2 | 3.9 | 1.6 |
|  | Difficulty | 24% | 4% | 8% | 15% | 18% | 19% | 8% | 4% | 3 | 5 | 4 | 2 | 4.1 | 1.5 |
| Capacity building in LMI settings: Training BME PhDs | Relevance | 17% | 3% | 1% | 7% | 8% | 11% | 15% | 38% | 5 | 7 | 6 | 2 | 5.6 | 1.7 |
|  | Maturity | 23% | 6% | 18% | 16% | 14% | 11% | 5% | 6% | 2 | 5 | 3 | 3 | 3.6 | 1.7 |
|  | Difficulty | 23% | 4% | 8% | 9% | 18% | 12% | 20% | 5% | 3 | 6 | 4 | 3 | 4.4 | 1.7 |
| Project-based learning in BME: Students learning while designing real medical devices | Relevance | 9% | 4% | 1% | 1% | 5% | 15% | 22% | 42% | 5 | 7 | 6 | 2 | 5.9 | 1.5 |
|  | Maturity | 13% | 9% | 15% | 12% | 23% | 19% | 5% | 3% | 2 | 5 | 4 | 3 | 3.6 | 1.6 |
|  | Difficulty | 13% | 4% | 11% | 11% | 16% | 26% | 14% | 6% | 3 | 5 | 5 | 2 | 4.3 | 1.6 |
| International medical design competitions, intensive hackathons and project-based learning summer/winter schools | Relevance | 14% | 5% | 2% | 9% | 5% | 16% | 18% | 30% | 4.5 | 7 | 6 | 2.5 | 5.3 | 1.8 |
|  | Maturity | 18% | 13% | 9% | 13% | 17% | 11% | 8% | 9% | 2 | 5 | 4 | 3 | 3.8 | 1.9 |
|  | Difficulty | 19% | 9% | 15% | 8% | 17% | 15% | 11% | 6% | 2 | 5 | 4 | 3 | 3.9 | 1.8 |

**Regulations and standards on medical devices**

|  |  | I don't  know | 1 (low) | 2 | 3 | 4 | 5 | 6 | 7 (high) | I  Quart. | III  Quart. | median | inter.  Range | mean | std |
| --- | --- | --- | --- | --- | --- | --- | --- | --- | --- | --- | --- | --- | --- | --- | --- |
| Harmonised regulations across LMI settings for medical technology development | Relevance | 5% | 4% | 1% | 5% | 6% | 14% | 26% | 39% | 5 | 7 | 6 | 2 | 5.7 | 1.6 |
|  | Maturity | 16% | 16% | 8% | 14% | 20% | 10% | 10% | 4% | 2 | 5 | 4 | 3 | 3.6 | 1.8 |
|  | Difficulty | 16% | 5% | 4% | 9% | 15% | 21% | 13% | 17% | 4 | 6 | 5 | 2 | 4.8 | 1.7 |
| Harmonised regulations across LMI settings for safe and ethical BME research | Relevance | 6% | 3% | 1% | 4% | 5% | 16% | 22% | 43% | 5 | 7 | 6 | 2 | 5.8 | 1.5 |
|  | Maturity | 20% | 9% | 16% | 10% | 18% | 15% | 7% | 5% | 2 | 5 | 4 | 3 | 3.7 | 1.7 |
|  | Difficulty | 18% | 6% | 7% | 13% | 11% | 18% | 15% | 13% | 3 | 6 | 5 | 3 | 4.5 | 1.8 |
| Harmonised regulations across LMI settings for adequate pre-market assessment | Relevance | 11% | 3% | 1% | 7% | 3% | 18% | 20% | 36% | 5 | 7 | 6 | 2 | 5.7 | 1.6 |
|  | Maturity | 27% | 12% | 10% | 19% | 9% | 11% | 4% | 7% | 2 | 5 | 3 | 3 | 3.5 | 1.9 |
|  | Difficulty | 24% | 7% | 4% | 8% | 13% | 21% | 9% | 13% | 3 | 6 | 5 | 3 | 4.5 | 1.8 |
| Harmonised regulations across LMI settings for adequate post-market surveillance | Relevance | 15% | 2% | 4% | 2% | 6% | 15% | 22% | 34% | 5 | 7 | 6 | 2 | 5.7 | 1.6 |
|  | Maturity | 29% | 14% | 11% | 10% | 13% | 16% | 3% | 4% | 2 | 5 | 3.5 | 3 | 3.4 | 1.8 |
|  | Difficulty | 24% | 5% | 9% | 5% | 11% | 17% | 11% | 18% | 3.5 | 6 | 5 | 2.5 | 4.7 | 1.9 |
| Publicly available standards, nomenclatures and databases on medical device products as alternative to privately developed documents: | Relevance | 7% | 3% | 3% | 4% | 7% | 10% | 13% | 52% | 5 | 7 | 7 | 2 | 5.9 | 1.7 |
|  | Maturity | 15% | 20% | 14% | 12% | 12% | 13% | 12% | 4% | 2 | 5 | 3 | 3 | 3.4 | 1.9 |
|  | Difficulty | 16% | 5% | 7% | 16% | 16% | 13% | 12% | 15% | 3 | 6 | 4 | 3 | 4.4 | 1.8 |
| Standards adequately addressing medical device usability in LMI settings | Relevance | 13% | 2% | 1% | 7% | 2% | 20% | 15% | 39% | 5 | 7 | 6 | 2 | 5.7 | 1.5 |
|  | Maturity | 24% | 9% | 14% | 16% | 12% | 14% | 7% | 4% | 2 | 5 | 3 | 3 | 3.6 | 1.7 |
|  | Difficulty | 22% | 6% | 9% | 6% | 19% | 19% | 8% | 11% | 3 | 5 | 4 | 2 | 4.3 | 1.8 |
| Standards adequately addressing medical device risk management in LMI settings | Relevance | 8% | 3% | 1% | 5% | 3% | 17% | 22% | 40% | 5 | 7 | 6 | 2 | 5.8 | 1.5 |
|  | Maturity | 22% | 8% | 10% | 19% | 15% | 14% | 8% | 4% | 3 | 5 | 4 | 2 | 3.7 | 1.7 |
|  | Difficulty | 17% | 5% | 11% | 10% | 18% | 20% | 4% | 14% | 3 | 5 | 4 | 2 | 4.3 | 1.8 |
| Standards adequately addressing the life-cycle of open source medical devices and technologies: | Relevance | 14% | 3% | 0% | 7% | 7% | 12% | 22% | 35% | 5 | 7 | 6 | 2 | 5.7 | 1.6 |
|  | Maturity | 25% | 10% | 9% | 22% | 11% | 9% | 8% | 4% | 2 | 5 | 3 | 3 | 3.6 | 1.7 |
|  | Difficulty | 20% | 6% | 11% | 7% | 18% | 16% | 10% | 12% | 3 | 6 | 4 | 3 | 4.3 | 1.8 |
| Standards adequately addressing point-of-care 3D printing of medical devices and technologies: | Relevance | 15% | 4% | 0% | 11% | 7% | 13% | 15% | 35% | 4 | 7 | 6 | 3 | 5.5 | 1.7 |
|  | Maturity | 32% | 14% | 16% | 14% | 12% | 9% | 2% | 2% | 2 | 4 | 3 | 2 | 3.0 | 1.6 |
|  | Difficulty | 27% | 5% | 14% | 7% | 12% | 15% | 10% | 11% | 2 | 6 | 4 | 4 | 4.2 | 1.9 |
| Standards adequately addressing privacy issues in innovative medical technologies: | Relevance | 11% | 6% | 1% | 10% | 5% | 16% | 17% | 33% | 4 | 7 | 6 | 3 | 5.3 | 1.8 |
|  | Maturity | 22% | 13% | 13% | 15% | 13% | 19% | 5% | 1% | 2 | 5 | 3 | 3 | 3.4 | 1.6 |
|  | Difficulty | 21% | 5% | 7% | 15% | 15% | 14% | 12% | 11% | 3 | 6 | 4 | 3 | 4.3 | 1.8 |

**Policy making and international partnerships**

|  |  | I don't  know | 1 (low) | 2 | 3 | 4 | 5 | 6 | 7 (high) | I  Quart. | III  Quart. | median | inter.  Range | mean | std |
| --- | --- | --- | --- | --- | --- | --- | --- | --- | --- | --- | --- | --- | --- | --- | --- |
| Policies pursuing capacity building in BME across LMI settings | Relevance | 18% | 0% | 3% | 7% | 10% | 12% | 13% | 37% | 5 | 7 | 6 | 2 | 5.6 | 1.5 |
|  | Maturity | 25% | 8% | 9% | 24% | 12% | 12% | 7% | 4% | 3 | 5 | 3 | 2 | 3.7 | 1.6 |
|  | Difficulty | 22% | 0% | 2% | 16% | 21% | 19% | 10% | 10% | 4 | 5.75 | 4.5 | 1.75 | 4.6 | 1.4 |
| Policies aimed at empowering the development of medical technology for LMI settings | Relevance | 14% | 2% | 2% | 2% | 9% | 17% | 16% | 38% | 5 | 7 | 6 | 2 | 5.8 | 1.5 |
|  | Maturity | 22% | 12% | 9% | 19% | 16% | 14% | 6% | 2% | 2 | 5 | 3 | 3 | 3.5 | 1.6 |
|  | Difficulty | 23% | 2% | 6% | 10% | 14% | 19% | 14% | 12% | 4 | 6 | 5 | 2 | 4.7 | 1.6 |
| Policies aimed at promoting the employment of open source medical devices | Relevance | 14% | 3% | 4% | 5% | 10% | 11% | 13% | 39% | 4 | 7 | 6 | 3 | 5.5 | 1.8 |
|  | Maturity | 26% | 17% | 8% | 19% | 13% | 12% | 1% | 4% | 2 | 4 | 3 | 2 | 3.2 | 1.7 |
|  | Difficulty | 23% | 4% | 5% | 11% | 11% | 24% | 10% | 11% | 3 | 6 | 5 | 3 | 4.5 | 1.7 |
| Policies aimed at fostering the employment of “zero kilometre” medical technologies* | Relevance | 17% | 6% | 3% | 2% | 13% | 18% | 9% | 32% | 4 | 7 | 5 | 3 | 5.3 | 1.8 |
|  | Maturity | 27% | 22% | 15% | 10% | 15% | 8% | 1% | 2% | 1 | 4 | 2.5 | 3 | 2.8 | 1.6 |
|  | Difficulty | 26% | 4% | 6% | 12% | 11% | 11% | 17% | 14% | 3 | 6 | 5 | 3 | 4.7 | 1.8 |
| Data-driven approaches for evidence-informed policy making and policy evaluation | Relevance | 14% | 3% | 4% | 2% | 9% | 15% | 17% | 36% | 5 | 7 | 6 | 2 | 5.6 | 1.7 |
|  | Maturity | 26% | 10% | 18% | 10% | 17% | 9% | 6% | 3% | 2 | 4.5 | 3 | 2.5 | 3.4 | 1.7 |
|  | Difficulty | 26% | 3% | 9% | 7% | 19% | 19% | 10% | 7% | 3.25 | 5 | 4 | 1.75 | 4.4 | 1.6 |
| International working groups monitoring good practices in BME for LMI settings | Relevance | 13% | 2% | 3% | 4% | 9% | 17% | 19% | 33% | 5 | 7 | 6 | 2 | 5.6 | 1.6 |
|  | Maturity | 26% | 7% | 17% | 17% | 16% | 11% | 3% | 3% | 2 | 4 | 3 | 2 | 3.4 | 1.5 |
|  | Difficulty | 20% | 5% | 6% | 14% | 14% | 18% | 13% | 10% | 3 | 6 | 5 | 3 | 4.4 | 1.7 |
| International working groups for monitoring global health issues | Relevance | 14% | 3% | 2% | 9% | 6% | 13% | 18% | 35% | 5 | 7 | 6 | 2 | 5.5 | 1.7 |
|  | Maturity | 23% | 7% | 9% | 15% | 17% | 12% | 9% | 7% | 3 | 5 | 4 | 2 | 4.0 | 1.8 |
|  | Difficulty | 21% | 6% | 5% | 15% | 16% | 18% | 7% | 11% | 3 | 5 | 4 | 2 | 4.3 | 1.7 |
| International working groups focused on matching needs and technologies | Relevance | 12% | 2% | 1% | 9% | 7% | 12% | 16% | 41% | 5 | 7 | 6 | 2 | 5.7 | 1.6 |
|  | Maturity | 28% | 7% | 7% | 15% | 17% | 18% | 4% | 4% | 3 | 5 | 4 | 2 | 3.8 | 1.6 |
|  | Difficulty | 21% | 5% | 6% | 11% | 21% | 16% | 11% | 9% | 3 | 5 | 4 | 2 | 4.3 | 1.7 |

* designed or manufactured in the point-of-care involving local populations):
